# Global Dialysis Perspective: Democratic Republic of Congo

**DOI:** 10.34067/KID.0000000000000303

**Published:** 2023-11-14

**Authors:** Ernest Kiswaya Sumaili, Yannick Mompango Engole

**Affiliations:** Renal Unit, Kinshasa University Hospital (KUH), University of Kinshasa, Kinshasa, Democratic Republic of the Congo

**Keywords:** arteriovenous access, arteriovenous fistula, chronic renal disease, chronic renal insufficiency, clinical epidemiology, daily hemodialysis, dialysis, ESKD, hemodialysis, hemodialysis access

## Introduction

CKD is an under-appreciated public health priority in the Democratic Republic of the Congo (DRC), despite a CKD prevalence of 12.4% in Kinshasa, the capital.^1^ Health care infrastructure is minimally developed, and most facilities lack basic services for CKD. Nephrologists are scarce, with only 38 nephrologists (32 adults and six pediatricians) in the country, comprising approximately 0.38 per million populations (pmp). In addition, because of the absence of efficient early detection and prevention of CKD programs in the country, many patients with CKD are diagnosed late, requiring dialysis on presentation. Dialysis is prohibitively expensive for the majority of Congolese people because there is no coverage for health benefits in either public or private.^2^

The DRC is a vast mineral-rich African country, but despite its wealth in natural resources, it remains one of the five poorest countries in the world. The natural resources are highly coveted by foreign entities, which drives the perpetuation of civil war in the East of the country. The massive displacement of populations resulting from the ongoing civil war, which certainly include hypertensives, diabetics, and patients having risk factors for CKD, exacerbates the burden of CKD because preventive measures or treatment of kidney disease risk factors are interrupted or discontinued. Here, we first describe how clinical nephrology in DRC is practiced and funded and then discuss opportunities to improve kidney care.

## Availability and Accessibility of KRT

A survey was administered to all existing hemodialysis centers from April 1 and May 15, 2023. Currently, there are only 263 patients on maintenance KRT in the country. Given the same prevalence of stage 5 CKD or 0.2% reported by Sumaili *et al.*^3^ in Kinshasa and by Masimango *et al.*^4^ in Bukavu at 2281 km from the capital city and with an estimated population of 98,124,583 (median age 17 years),^5^ the estimated prevalence of treated KRT should, however, be more than 98,100.

**Figure 1:**
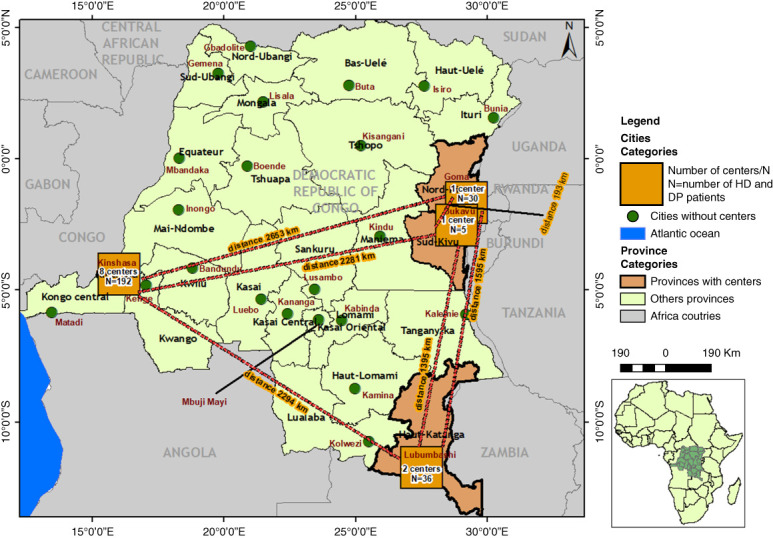
**Distribution of the existing hemodialysis centers in the DRC.** The map depicts the 26 provinces of DRC detailing the current number of patients on maintenance hemodialysis and the number of centers in each province and the distance between the provinces. The provinces labeled in green lack hemodialysis centers. DRC, Democratic Republic of the Congo.

Hemodialysis is the most common form of KRT (98.4%), followed by continuous ambulatory peritoneal dialysis (PD) (1.5%). Transplantation is not available. Of the 26 provinces, only five provinces have hemodialysis centers (Figure 1). Table 1 highlights the regional distribution of hemodialysis and PD patients combined. Although, there are four PD centers, only the Kinshasa University Hospital has four patients on continuous ambulatory PD. The three other PD centers located in Kinshasa (Pediatric Department of Kinshasa University Hospital), Kisantu (Kongo central), and Bukavu (South-Kivu) only provides PD for AKI in children, as part of saving young lives program.^6^ Figure 1 illustrates the discrepancy in availability of kidney care between Kinshasa and the provincial cities. Numbers of hemodialysis centers and doctors are highest in cities, with better living standards and higher incomes.

**Table 1:** Dialysis data of dialysis (hemodialysis and peritoneal dialysis) centers in the Democratic Republic of the Congo

Variable	Whole Group *N*=263	Nonprofit *n*=90	Profit Unit *n*=173	*P* Value
Type of dialysis				0.005^a^
Center hemodialysis	259 (98.4)	86 (95.5)	173 (100)	
CAPD	4 (1.5)	4 (4.0)	0	
Home hemodialysis dialysis	0	0	0	
Causes of ESKD				0.116
Hypertension	73 (27.7)	20 (11.5)	53 (30.6)	
Diabetes	63 (23.9)	18 (10.4)	45 (26.0)	
CGN	44 (16.7)	15 (8.6)	29 (16.7)	
Obsturo	21 (7.9)	5 (2.8)	16 (9.2)	
ADPKD	13 (4.9)	2 (1.1)	11 (6.3)	
HIV	4 (1.5)	1 (0.5)	3 (1.7)	
Others	45 (17.1)	29 (16.7)	16 (9.2)	
Cities				<0.0001^a^
Kinshasa	192 (73.0)	81 (90.0)	107 (61.8)	
Lubumbashi	36 (13.6)	0	36 (20.8)	
Goma	30 (11.4)	0	30 (17.3)	
Bukavu	5 (1.9)	5 (5.5)	0	
Age (years at initiation)	50.1±6.4	52.5±3.1	48.9±7.5	0.153
% male	188 (72.5)	64 (71.1)	124 (71.6)	0.932
Vascular access				
AVF	40 (15.4)	8 (8.8)	32 (18.4)	0.039^a^
CVC	219 (84.5)	82 (89.9)	141 (81.5)	
AVG	0	0	0	
Average length of dialysis	4	4	4	
Patient/nurse ratio	1:4	1:3	1:4	
Session				0.063
1×/wk	31 (11.9)	14 (15.5)	19 (10.9)	
2×/wk	91 (35.1)	33 (36.6)	60 (34.6)	
3×/wk	137 (52.8)	38 (42.2)	94 (54.3)	
Cost/session USD				
Hemodialysis	141.6±41.0 (80–200)	141.2±48.0 (100–175)	141.8±40.7 (80–200)	0.437
PD	80 per day	80 per day	—	
Out of pocket	144 (59.2)	59 (65.5)	85 (49.1)	0.011^a^
Employer/government	100 (23.2)	23 (25.5)	77 (44.5)	
Insurance coverage	19 (7.2)	8 (8.8)	11 (6.3)	
Reimbursement	8 (3.0)			

^a^*P*<0.05.

Data are expressed as absolute (*No.* ) proportions or relative frequency (%) and mean±SD. ADPKD, autosomal dominant polycystic kidney disease; AVF, arteriovenous fistula; CVC, central venous catheter, AVG, arteriovenous graft; CGN, chronic GN; CAPD, continuous ambulatory peritoneal dialysis; Obstructive uropathy, Obsuro; PD, peritoneal dialysis.

The number of dialysis patients has dropped significantly between the present survey and that of Nlandu e*t al.* in 2021^7^ (360–263), contrasting with an ever-increasing number of hemodialysis centers (around 24). The reasons for this include the prohibitive out-of-pocket costs leading to deaths due to withdrawal from dialysis, relocation of some patients abroad, and renal transplantation (performed abroad). Some patients died when their philanthropic (nonprofit) low-cost center closed in 2019 because of financial constraints, and they were unable to afford hemodialysis at other, more expensive centers. The ongoing war, as well the natural disasters in the East of the DRC, contributes to mortality by preventing the supply of consumables to rebel-occupied areas or preventing patients from getting to dialysis centers. Electricity and water cuts also hamper to smooth running of the centers.

The average age of hemodialysis patients in the current survey of 50 years at initiation of dialysis corroborates our previous study,^1^ highlighting that CKD affects younger adults in Africa compared with high-income countries. The causes of CKD are hypertension (27.7%), diabetes (23.9%), and chronic GN (16.7%). Hence, CKD in DRC reflects the double burden of communicable and noncommunicable diseases.^1,8^ Men predominate among Congolese hemodialysis patients, reflecting the generally chauvinistic society.

## Management and Funding of KRT in the DRC

Most of the 263 prevalent patients are undergoing hemodialysis. Overall, 15.4% of patients have arteriovenous fistulas (more common in for-profit than in nonprofit units (18.4% versus 8.8%, *P* = 0.03, Table 1). Factors which may explain this low arteriovenous fistula prevalence include the shortage of vascular surgeons, high cost, practitioner inertia, and negative influence of neighboring patients (aesthetic, time for hemostasis). The remainder (85%) use catheters (62% tunneled and 22.6% temporary catheters). The average length of dialysis is 4 hours. Patients who can afford it dialyze 3 times a week (52.8%), and 12% and 35.1% undergo hemodialysis once and twice weekly, respectively. Nephrologists mostly see patients in every dialysis session. Dialysis is delivered by nurses (Table 1). Dialyzers are not reused. Mokoli *et al.*^9^ in 2016 reported a median survival of 17 months among 250 hemodialysis patients, with a cumulative survival of 84.8% at 3 months, 71.6% at 6 months, 62.8% at 1 year, and 57.2 at 4 years.

Access to dialysis in the DRC is severely limited by insufficient infrastructure, geographic inaccessibility, and catastrophic out-of-pocket expenses. Most patients with kidney failure die. Palliative care is still nascent. The average direct cost of a hemodialysis session is USD 141.6±41, with no significant difference between the private (profit) and nonprofit units (Table 1). It is unclear why the cost of hemodialysis does not differ between private and state centers, as occurs elsewhere, for example, in Haiti (USD 20 in the state university hospital versus USD 200 the private sector).^10^ Given the absence of health insurance and lack of government subsidies to cover the high costs of KRT, dialysis companies lease dialysis machines and consumables in the private and nonprofit units, charging USD 60–100 per hemodialysis session and providing a set number of 40–60 bundles per month. Over time, if centers remain loyal to the bundle contract, the machines will belong to the centers, which should reduce their costs. There is, however, one monopoly importer of medical devices, which buys what it wants (often second-hand) and sets huge prices. In addition, hemodialysis machines sometimes carry codes granting exclusivity of consumables to one company. The clear lack of regulation of the dialysis market in the DRC contributes to the exorbitant cost of hemodialysis.

## Perspectives

To tackle the burden of CKD in the DRC, prevention must be prioritized. Hence, nonphysician health care workers and general physicians should be engaged in this preventive effort. A multisectoral approach should include better health education of the general population and the prevention of not only noncommunicable diseases (hypertension, obesity, diabetes *etc.*) but also communicable diseases (malaria, HIV, hepatitis). Dialysis services should be developed as part of an integrated KRT program, not solely to increase access but also with the goal of kidney transplantation for suitable patients. Regulation of dialysis is needed to control costs. The government and its partners should invest in putting an end to this unjust civil war and strengthening health systems to cope with natural disasters and prepare for the unexpected. Realization of universal health coverage should improve general health and will contribute to reducing the burden of kidney diseases. To achieve the universal health coverage, adequate health financing, peace, governance, and effective leadership are required.
